# Long-term serotonin abnormalities in the brain of immature rats subjected to febrile seizures

**DOI:** 10.22038/IJBMS.2023.70273.15297

**Published:** 2023

**Authors:** Omnia Ashoor, Haitham S. Mohammed, Nasr M. Radwan, Reem Elge-baly

**Affiliations:** 1 Biophysics Department, Faculty of Science, Cairo University, Giza, Egypt; 2 Zoology Department, Faculty of Science, Cairo University, Giza, Egypt

**Keywords:** Cortex, Febrile seizures, Hippocampus, Hyperthermia, Monoamines, Rat

## Abstract

**Objective(s):**

Febrile seizures (FS) are the most common neurological disorder at a young age in humans. Animal models of hyperthermia-induced seizures provide a tool to investigate the underlying mechanisms of FS related to epilepsy development and its co-morbidities. The present study investigates the alterations in monoamine neurotransmitters in two brain areas: the cortex and the hippo-campus in animals subjected to prolonged FS at their immature age.

**Materials and Methods:**

Experimental animals were divided into three groups: cage-control group (NHT-NFS), positive hyperthermic control group (HT-NFS), and the hyperthermia-induced febrile seizure group (HT-FS). Each group was further subdivided into young (Y) and adult (A) groups.

**Results:**

There were significant changes in the cortical and hippocampal serotonin neurotransmitters that were persistent until adulthood. However, the changes in the two other neurotransmitters, norepinephrine and dopamine, were transient and have been recovered in adulthood.

**Conclusion:**

The present study sheds more light on the importance of monoamine neurotransmitters in epileptogenesis following FS.

## Introduction

Febrile seizures (FS) are convulsions triggered by fever. They occurs most frequently in childhood. Nearly 5% of children between the ages of six months and five years would experience FS, with a peak incidence occurrence of around 1.5 years (1). Based on seizure duration, physical characteristics, and recurrence patterns, FS can be categorized as either simple or complex. Seizures are classified as generalized, focal, or of unknown onset, with subcategories of motor, non-motor, and with retained or impaired consciousness for focal seizures, according to the International League Against Epilepsy (ILAE) reclassification in 2017 (2). Usually, a simple seizure lasts from a few seconds to 15 min and is characterized by a rapid recovery with no adverse consequences (3). Complex FS, on the other hand, frequently have one or more of the following characteristics: seizure recurrence within 24 hours of the index febrile illness; postictal neurological impairments, focal features, prolonged duration (more than 15 min) (4)

The cause of FS is multi-factorial, in conjunction with the underlying genetic predisposition and environmental causes. FS is typically thought to result from the vulnerability of the developing central nervous system (CNS) to fever (5). Even though FS are usually benign, young children who had FS have a fivefold increase in FS compared to the unaffected ones. Seven percent of children who experience a febrile seizure will also experience an afebrile seizure at the age of 25. A small fraction of children who experience long-lasting FS eventually experiences mesial temporal lobe epilepsy. FS are perfect examples of the higher propensity for seizures in developing brains. Adults rarely experience seizures as a result of fever, and 1–14 % of febrile seizure cases result from fever. (6, 7)

Animal models of FS have been utilized to elucidate the underlying mechanisms of FS, investigate their link to the development of temporal lobe epilepsy (TLE), and search for a suitable intervention to interfere with the epileptogenesis process (8-10). Animal models of FS that are based on hyperthermia-induced seizures are gaining validity since some symptoms that were documented in children are successfully manifested in hyperthermic FS models (11). Hyperthermia-induced seizures in rats are age-dependent, in young rats (7 – 12 days) body temperatures of 40–42 °C are sufficient to trigger seizures (12, 13), while a body temperature of 42–44 °C may be required to induce a seizure in older rats (22–29 days) (14).

Most animal models of FS are carried out at the age when significant hippocampal development takes place. This occurs in rats in the second postnatal week, which is thought to be parallel to the first year of human life (15). Studies are controversial as to whether the early-life FS contributes to adulthood epilepsy and spontaneous seizure induction. Most studies have focused on the development of limbic seizures to better understand the role of TLE development in humans after FS (1). Spontaneous limbic seizures have been shown in 35% of animals three months after FS with the model of hyperthermic seizures, with inter-ictal epileptiform discharges in 88%. The causal link between prolonged FS and TLE is clinically important, because predictive biomarkers and preventive therapies may be feasible if this link exists (16).

It has been suggested by a large body of experimental and clinical evidence that monoamines could play an important role in regulating epileptogenesis, convulsions, susceptibility to seizures, and psychiatric disorders that are common co-morbidities in people with epilepsy (17). However, due to the complexity of these neurotransmitter systems, neither the relative importance of individual monoamines nor their interaction has been thoroughly explained yet. Some studies have emphasized the role played by monoamines in epilepsy by showing that monoamine-dependent disorders, such as depression and other neuropsychiatric disorders, could elevate the risk of seizures (18, 19). On the other hand, some research revealed that depression is a key predictor of seizure complexity. (20, 21). 

Kilian and Frey (22) evaluated the role of central monoamines in the animal model of epilepsy and concluded that a certain role is played by serotonin and noradrenaline in determining the threshold of seizures in animals. It was reported that external or internal disturbances in very young brains are likely to hinder biogenic amine synthesis (21). 

Serotonergic neurotransmission regulates a wide range of experimentally induced seizures and is thought to be implicated in the increased seizure susceptibility seen in rodents with a hereditary predisposition to epilepsy (23). Norepinephrine (NE) is a neurotransmitter that is produced by noradrenergic terminals principally in the locus coeruleus (LC), which then delivers NE-containing axon projections to a variety of brain locations, including those implicated in epilepsy (24). The functions of dopamine (DA) in the body span from voluntary movement control to hormone and blood pressure regulation. Since the 1960s, DA’s seizure-modulating effects have got a lot of attention. Bozzi and Borrelli (25) addressed the intracellular signaling pathways induced by the activation of several DA receptors (DARs) in connection to their function in limbic seizures and epileptogenesis (25).

The present work aims to investigate the variations in the monoamine neurotransmitter levels in the cortex and hippocampus of an animal model of FS and the long-lasting and persisting changes in the adult animals that have been subjected to FS at a young age. The ultimate goal of the present investigation is to provide some basic knowledge that can be used later in developing a suitable intervention that ceases the suffering of epilepsy patients. 

## Materials and Methods

***Ethical approval***

All experiments were done according to the international guidelines of animal care and use and were approved by the local Committee of animal care and Use (ACUC) under the number (CU/I/F/35/18).

***Experimental animals***

Wistar rats were supplied by the National Cancer Institute, Giza (Egypt). The adult rats were kept for 14 days to acclimatize to the laboratory conditions with a 12 hr light / 12 hr dark cycle. Standard diets and water were available *ad libitum*. Animal breeding was conducted in the animals’ house facility at the Faculty of Science, Cairo University. The day of the neonate’s birth was recorded for each rat and is considered day zero (P0) after birth. At P14, the gender of the young rats was determined, and male rats were selected for the present study. 

***Experimental design***

**Figure 1:**
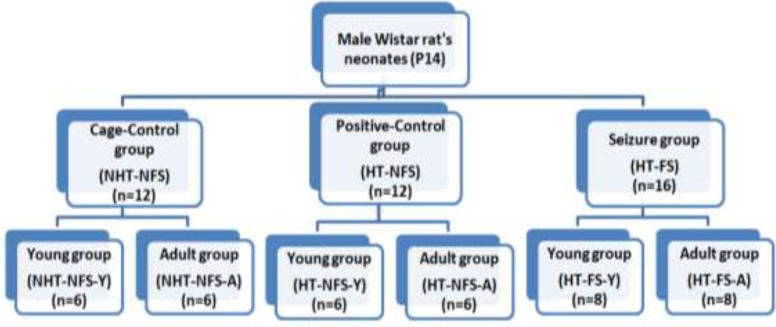
Diagrammatic representation of the male Wistar rats experimental design of cage-control, positive control, and seizure groups with number of animals between brackets

The total number of experimental animals was (n = 44), which were divided into three groups (Figure 1). The brain of each animal was dissected into cortex and hippocampus samples.

 The hyperthermia-induced febrile seizure (HT-FS) (n=16) consisted of the animals which displayed the tonic-clonic complex FS (>15 min), the positive control group (HT-NFS) (n=12) consisted of the animals exposed to HT and did not display convulsive seizure, and the cage-control group (NHT-NFS) (n=12) consisted of the animals were not expose to hyperthermia (HT). The number of animals that died during HT was recorded (n = 4). The exact reason for these animals’ mortality was unknown except they may be sensitive to hyperthermia. The HT exposed and control groups were further subdivided into a young group (Y) which was investigated at a young age, and an adult group (A) which was investigated in their adulthood (after 3 months of HT). All animals were monitored during the experiments and the criteria for humanely euthanizing an animal before the experimental objective is achieved were marked weight loss, ulcerative skin lesions, and any uncontrollable aggressive behavior. However, none of these criteria have been displayed by the experimental animals included in the present study.

To avoid the impact of animal selection bias on the experimental results, animals were allocated to the experimental groups in a randomized method. There was no blinding during the conduct of the experimental work, however, proper blinding has been implemented during data measurements and analysis.

***Hyperthermia and seizure induction***

Experimental FS was induced in neonatal rats on the 14^th ^post-natal day (P14) by using the hot air model, which was developed by Baram and coworkers (16, 26) and described earlier by Crespo *et al***. **(27, 28). Hyperthermia (HT) was carried out in a 1-liter glass beaker covered from the bottom by 3 layers of paper to prevent unfavorable heating and burning from the glass itself. So, the only source of heating was the adjustable flow of the heated air coming from the hot air source. The vertical distance between the bottom of the beaker, where the animal is, and the source of hot air was adjusted every trial till the ambient temperature reached 50 ± 1 °C.

The initial rectal (core) temperature and weight of each animal were recorded before the start of the HT. Rectal temperature was recorded every 2 min using a thermocouple temperature probe of the model (TP-02) connected to a digital multimeter model (KYORITSU, KEW1011, Japan). HT was maintained for 30 min or till the core body temperature of the young rodents reaches 40-44 °C. The behavior of the animal was monitored during this period of HT. If the animal was exposed for 30 min to HT or its core body’s temperature reached 40–44 °C without experiencing a seizure, the process of HT was stopped, and the animal was excluded from the FS group. Seizure onset was characterized by biting and chewing (facial myoclonus) behavior and falling over accompanied by hind-limb clonus movements (stage 5 on the Racine scale) (29). The complex seizure was determined by tonic-clonic convulsions that continued in animals for more than 15 min, and those animals were grouped as the FS group (HT-FS) (n=16). Cage-control animals** (**NHT-NFS) (n= 12) were isolated from their mothers for the same time taken in the HT process. The non-seizure hyperthermic animals (HT-NFS) (n=12) were the animals exposed to HT without displaying any feature of seizures and these animals served as a positive control group. At the end of each HT process, neonates were immediately transferred to a cool surface till their temperature returned to normal (initial) core temperature and then returned to their mothers.

***Brain dissection and tissue sample collection***

At the end of each experiment, animals were sacrificed by sudden decapitation. The advantage of using swift decapitation is to obtain brain tissues that are not contaminated or functionally changed by gases and anesthetic. This procedure is considered an acceptable humane euthanasia procedure (30). After dissecting the brain from the skull on an iced dish, the cortex and hippocampus were separated from the brain. Collected samples were weighed and stored in a deep-freezer (-30 °C) till the time of the measurements.

***Determination of monoamines in samples by spectrofluorometry***

In an ice-cold solution of acidified n-butanol (cat. no.281549, Sigma Aldrich), each rat’s cortex and hippocampus were homogenized. Every brain region’s homogenate was centrifuged for 5 min at 2000 rpm. The supernatants were used according to the fluorometric method defined by (31, 32) to estimate Dopamine (DA), Norepinephrine (NE), and serotonin (5-HT). A spectrofluorometer (model Jasco-FP-6500, Japan) was used to measure the fluorescence with a 150 W xenon arc lamp source (excitation slit bandwidth of excitation monochromator 5 nm, emission slit bandwidth of emission monochromator 5 nm).

***Serotonin (5-HT) level determination***

Three 5-HT standards were formulated in duplicate and have achieved a total volume of 0.2 ml for all 5-HT tubes, plus a blank reagent (0.2 ml of 0.2-N acetic acid, cat no.45754, Sigma Aldrich),1.2 ml OPT was added and mixed (OPT reagent consisted of 20 mg% in cont. HCl, cat no.H1758, Sigma Aldrich). All tubes were left in a boiling water bath for 10 min, then cooled with tap water, and read by spectrofluorometer (excitation wavelength: 355 nm and emission wavelength: 470 nm).

***Norepinephrine and dopamine levels determination***

The standards for NE and DA were prepared using three different concentrations in duplicate with 0.2-N acetic acid reaching a total volume of 1.6 ml per tube. Five milliliter of heptane (cat no.34873, Sigma Aldrich) and 2.5 ml of acidified n-butanol were added to the tubes, mixed, and centrifuged for 5 min at 1000 rpm. The organic supernatant phase was aspirated, discarded, and 1 ml of the aqueous layer was transferred to tubes. To all tubes (standards, reagent blank, and samples), 0.2 ml of EDTA (cat no. E7889, Sigma Aldrich) was added and mixed. 0.2 ml of alkaline sulfite (cat no.407410) was added to the reagent blank (1 ml of 0.2-N acetic acid), followed by 0.1 ml of 0.1 N iodine (cat no.1.09099, Titriputreag. Ph, Eur) and 0.2 ml of 5-N acetic acid. To all remaining tubes 0.1 ml of 0.1-N iodine was added, followed by 0.2 ml of alkaline sulfite added after 2 min. Two min later, 0.2 ml of 5-N acetic acid was added and mixed. All tubes were placed for 2 min in a boiling water bath, cooled using tap water, and read for NE fluorescence. Excitation was made at wavelength 380 nm, and emission was measured at wavelength 460 nm. All solutions were returned to their original test tubes and re-heated in a boiling water bath for an additional 5 min and cooled under tap water. For DA, excitation and emission wavelengths were 320 and 375 nm, respectively.

***Calculations of monoamine concentration***

Fluorescence units for standards were graphed (after subtracting the appropriate blank values) with fluorescence units as a function of concentration. Values for samples were derived from this graph and using the simple proportion:

$X=\frac{\mathrm{Sample of weight in \mu g}\times 1000}{\mathrm{Weight of the brai}n^{'}\mathrm{stissue sample}}$

Where X is the concentration of monoamine in μg/g wet brain tissue.

***Statistical analysis***

Deciding whether to use a parametric or nonparametric test depends on the normality of the data. Therefore, the first step was to check the data normality and variance homogeneity. Using the Statistical Package for Social Sciences (SPSS) we investigated the following outputs: The Shapiro-Wilk p-value, which was above 0.05. The Skewness and kurtosis z-values were between -1.96 and +1.96. The Histograms and normal Q-Q plots indicated that our data are normally distributed.

The data is presented in mean values ± standard deviation. One-way analysis of variance (ANOVA) using SPSS software was used to test statistical significance between groups followed by a *post hoc* test (Duncan test) to compare significance between group pairs at *P*-value<0.05. The percentage difference was determined using the following formula. 

## Results

***Febrile seizure threshold and latency***

**Figure 2:**
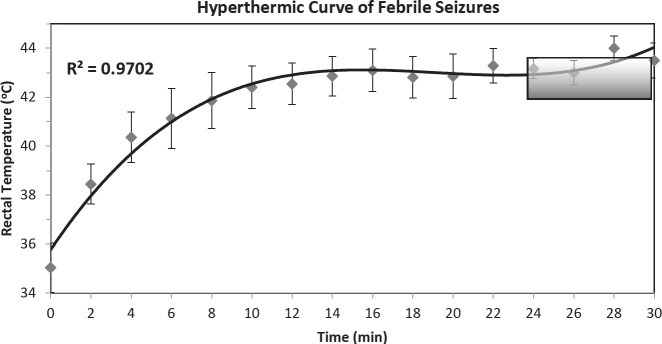
Animal’s rectal temperature variation with hyperthermia, n=16

Determination of the relationship between core body temperature, threshold, and latency for seizure initiation is necessary to describe the dynamic nature of seizures induced by hyperthermia and for any interventions that could be developed. As shown in Figure 2, the average baseline (normal) rectal temperature of the experimental animals at P14 was 35.04± 1.01 °C (n = 20 pups). The tonic-clonic febrile seizure was initiated at an average threshold temperature of 43.27 ± 0.63 °C. While the average latency for seizure initiation was 26.82 ± 3.01 min including the time interval of one min heating off every two min, and the average seizure duration was 19.25± 3.58 min. As shown in Figure 2, the relation between the time of the hyperthermia and the increase in the core body temperature was non-linear. There was a rapid increase in animal core temperature at the start of hyperthermia followed by a slower increase that was followed by a semi-constant (plateau) core temperature. The relation between the hyperthermia time and the core body temperature can be expressed mathematically by the following polynomial equation:

y = -0.0143x^2^ + 0.6185x + 37.043

Where y is the animal core temperature in Celsius degrees and x is the time of hyperthermia exposure in minutes. 

***Cortical level of monoamines in young rats***

**Figure 3:**
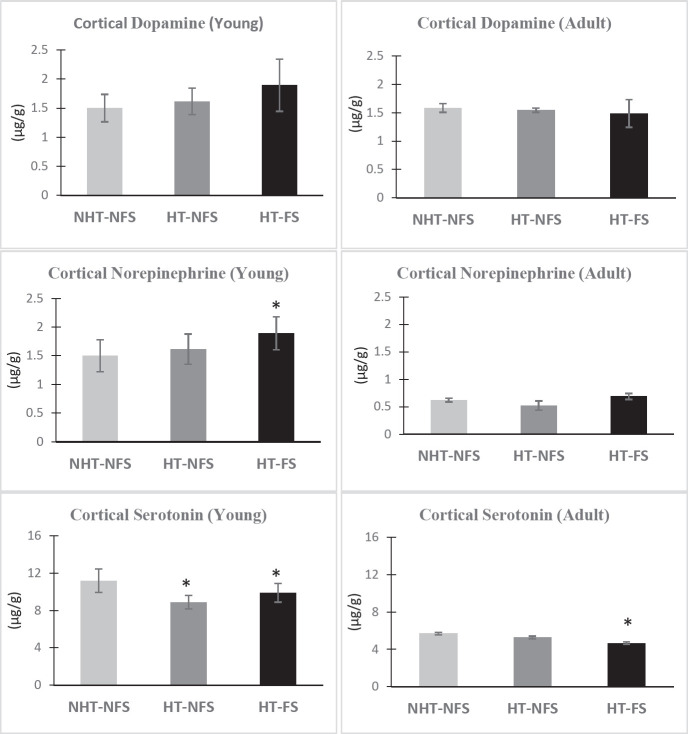
Variations in the monoamine levels (µg/g) in the cortex of young (Y) and adult (A) groups of rats

Determination of monoamine neurotransmitter levels in certain brain areas in hyperthermia-treated animals and comparing these values with the control group of animals provide data that are related to the disturbance occurring in these important neurotransmitters and this, in turn, could interpret the impairments that may evolve as a consequence of these abnormalities. As shown in Figure 3, in the HT-FS group of young animals, the level of cortical NE was significantly increased by (37.35%, F (2,17) = 3.834, *P*=0.042) compared to the cage-control group, while the 5-HT level significantly decreased by (-11.55%, F (2,17) = 7.716, *P*=0.004) compared to the cage-control animals (NHT-NFS, Figure 3). On the other hand, DA was non-significantly increased in the HT-FS group compared to the other groups. While in the non-seizure hyperthermic group, DA was slightly increased and there was a significant reduction in the level of 5-HT by (-20.46 %, F (2, 17) = 7.716, *P*=0.004) compared to NHT-NFS group. 

***Cortical level of monoamines in adult animals***

For adult groups, analysis of variance revealed that DA and NE in the cortices of the adult animals were slightly and non-significantly changed in the different groups related to the NHT-NFS group. On the other hand, there was a significant decrease in 5-HT levels by (-17.98%, F (2,17) = 13.473, *P*=0.0001) in HT-FS animals compared to the NHT-NFS group (Figure 3).

***Hippocampal level of monoamines in young rats***

**Figure 4:**
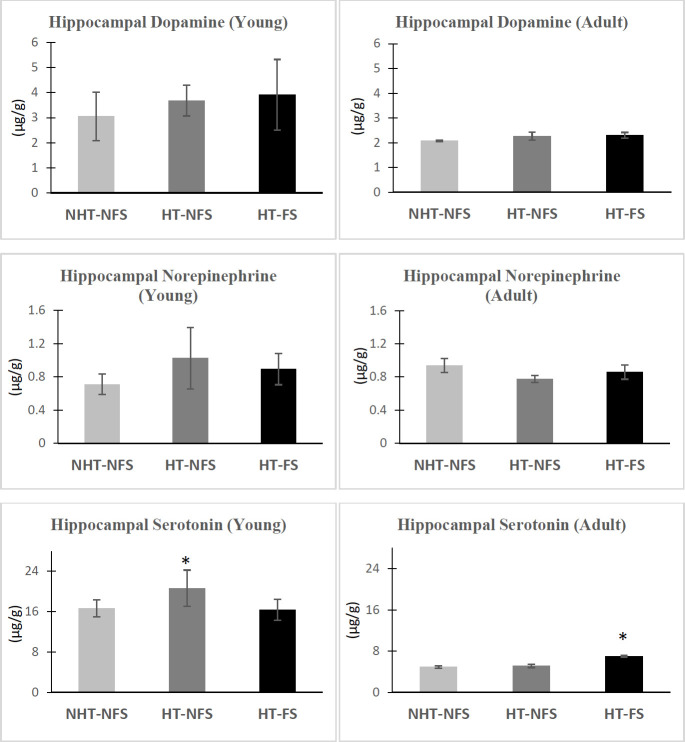
Variations in the monoamine levels (µg/g) in the hippocampus of young and adult groups of rats

As shown in Figure 4, analysis of variance revealed that DA in the hippocampus of the young animals was only slightly and non-significantly changed in the different groups, however, a slight increase in its level was recorded in the HT-FS group compared to all other groups. On the other hand, in the HT-NFS group, there was a significant increase in the hippocampal level of 5-HT by (23.85%, F (2,17) = 5.651, *P*=0.013), compared to the NHT-NFS group. In the HT-FS group, DA, NE, and 5-HT levels recorded non-significant changes compared to the NHT-NFS group.

***Hippocampal level of monoamines in adult animals***

Analysis of variance revealed that in the HT-FS group, there was a slight increase in DA level and a slight decrease in NE level compared to the NHT-NFS. However, it is found that 5-HT level increased significantly in that group with 42.30% (F (2,17) = 25.155, *P*=0.0001, Figure 4). On the other hand, non-significant changes have been obtained in the hippocampal levels of DA, NE, and 5-HT in the HT-NFS group compared to the NHT-NFS group.

NHT-NFS: non-hyperthermic and non-febrile seizure group, HT-NFS: hyperthermic and non-febrile seizure group, and HT-FS: hyperthermic and febrile seizure group. (A): adult an-imals and (Y): young animals

**Table 1:** Initial sample size of male adult Wistar rats in each group, and the number of mortality

Group	HT-FS	HT-NFS	NHT-NFS
No. of animals	20	12	12
No. of mortality	4	0	0

HT-FS: hyperthermic and febrile seizure group; HT-NFS: hyperthermic and non-febrile seizure group; NHT-NFS: non-hyperthermic and non-febrile seizure group

The dark rectangle represents the average time and temperature of hyperthermia-induced seizures. Data was repre-sented in mean ± SD

NHT-NFS is the cage-control group that was not exposed to hyperthermia nor had febrile seizures (n=6 for each group from A&Y), HT-NFS is the hyperthermia-exposed animals that did not suffer from febrile seizures (n=6 for each group from A&Y), and HT-FS is the hyperthermia-exposed animals that suffered from febrile seizures (n=8 for each group from A&Y). The data is presented in mean values ± standard deviation. One-way analysis of variance was used to test statistical significance between groups followed by a *post hoc *test (Duncan test) to compare significance (*) between group pairs at *P-*value<0.05

NHT-NFS is the cage-control group that was not exposed to hyperthermia nor had febrile seizures (n=6 for each group from A&Y), HT-NFS is the hyperthermia-exposed animals that did not suffer from febrile seizures (n=6 for each group from A&Y), and HT-FS is the hyperthermia-exposed animals that suffered from febrile seizures (n=8 for each group from A&Y). The data is presented in mean values ± standard deviation. One-way analysis of variance was used to test statistical significance between groups followed by a *post hoc* test (Duncan test) to compare significance (*) between group pairs at *P*-value<0.05

NHT-NFS: non-hyperthermic and non-febrile seizure group; HT-NFS: hyperthermic and non-febrile seizure group; HT-FS: hyperthermic and febrile seizure group

## Discussion

The present study investigated the neurochemical changes that took place in the cortex and hippocampus of young and adult rats subjected to premature hyperthermia-induced seizures. The main aim of the present work focused on the persistent changes in cortical and hippocampal neurotransmitters.

Hyperthermia-induced FS in animals have been utilized to study the underlying mechanisms of seizure’s effects on the brain and to find an association between early-life FS and the development of TLE later in life (10). FS induced by hyperthermia in animal models displays symptoms that are well-documented in humans. Hyperventilation-induced alkalosis, freezing, and oral automatisms are among the symptoms shared between human and rodent models of FS (33).

In the present work, hyperthermia was utilized to induce FS in young immature rats. The core body temperature of the animals displayed two phases: the initial phase where body temperature was raised quickly, and then plateaued or small changes occurred during the second phase despite the continued exposure to the hot air. The FS was triggered in animals during the period when the curve plateaued. These findings are in agreement with the study of Scantlebury and coworkers. They documented that the exposure to hot air resulted in an initial fast elevation in core temperature followed by a plateau (34). The curve of the core body temperature variation with time can be represented by a polynomial function (35).

Accumulated evidence has been recently directed towards the role played by neurotransmitter systems and in particular monoamines in seizure susceptibility, convulsions, the process of epileptogenesis, and the psychiatric co-morbidities seen in epileptic patients (19). Therefore, investigating the disturbance in the neurotransmitter systems, accompanied by seizures and convulsions, is an important task to explain this relationship. In the present study, the variation in monoamine levels in the cortex and hippocampus was investigated in the experimental animal model of FS. These investigations were carried out in young rats and in adult rats that have been subjected to FS during their developing age. 

Biogenic amines are some of the most common categories of neurotransmitters in the rat’s brain and it consists of serotonin (5-HT) and the catecholamines, dopamine (DA) and norepinephrine (NE) (20). 

The present results showed a significant decrease in the cortical 5-HT in young HT-FS animals which has persisted to be shown in the adult HT-FS group compared to the NHT-NFS group of animals. On the other hand, a significant increase has been obtained in the hippocampal 5-HT levels in the adult HT-FS group compared to the NHT-NFS group. The alteration in the serotonergic neurotransmission is a common feature in patients that suffer from depression and epilepsy (36). The large number and variety of different receptors that interact with 5-HT and are linked to divergent signaling pathways emphasize the complex and important role played by this neurotransmitter in the modulation of brain excitability and function (37). It was reported that 5-HT receptor subtypes are differently distributed in different brain areas and circuits that could be involved in epilepsy (37). Furthermore, the important role played by 5-HT in the development of the cortical tissue’s structure and function is well documented (38). A decline in cortical 5-HT has been reported in different animal models of epilepsy and has been attributed to the decrease in the receptor binding and the increase in the receptor density of cortical 5-HT (37, 39). Therefore, the significantly attenuated levels of the cortical 5-HT in the young and adult FS groups could be a manifestation of the seizure activity that hinders the proper development of the cortical tissue.

The significant elevation in the hippocampal 5-HT obtained in the present study in the adult FS group could be interpreted from the perspective of a compensatory effect. The increase in the extracellular level of 5-HT could have an anti-seizure effect in different animal models of epilepsy (37, 40). However, the direct and obvious indication that can be deduced from these significant changes recorded in the level of 5-HT level in the cortical and hippocampal tissues of the adult HT-FS group is that early-life induced FS is contributing to the disturbance in neurotransmitter systems in adulthood. 

A significant elevation in the hippocampal 5-HT level has been obtained in the present study in the positive control animals that were subjected to hyperthermia and did not develop seizure activity (HT-NFS). This finding could be explained as a transient effect of hyperthermia which was found to disrupt the blood-brain barrier (BBB) and temporarily allows the entrance of the peripheral 5-HT (41)**.** The transient nature of this effect has been confirmed from the determination of 5-HT level in the adult animals of the corresponding group (none-seizure group) that showed only non-significant elevation in the hippocampal 5-HT level. 

In the present study, there was a significant elevation in the level of NE in the HT-FS young group of animals and this elevation disappeared in the adult group, which means that this effect is recovered and cannot be considered a persistent change. The direct relation between NE and stress could explain this elevation in its cortical levels. Both hyperthermia and epileptic seizures could be considered stressors that may give feedback on the NE system and lead to an increase in NE levels (42). This finding is in line with clinical data that reported higher CSF levels of NE in epileptic patients when compared with controls (43). Confirming the relationship between NE and stress induced by seizures in young animals is the absence of significant changes in this neurotransmitter in adult groups in the present investigation. 

Despite the insignificant changes in DA levels in young and adult animals compared to the cage-control group, the importance of DA in FS and epilepsy ca not be ignored. Frosini and his coworkers reported that all excitatory and inhibitory neurotransmitters are elevated by hyperthermia, and this elevation in neurotransmitters may be attributable to their essential role in thermal control (44). The increased secretion of catecholamines (DA and NE) is also known as an endocrine response to hyperthermia (45). Thus, the present increase in the cortical and hippocampal DA in young animals may be due to the action of hyperthermia, and when animals reached the adultness stage, this effect disappeared and returned to normal.

Alteration in the monoamine neurotransmitters obtained in the present study emphasizes the involvement of monoaminergic systems in the pathogenesis of epilepsy. It emphasizes that FS children are at greater risk of a wide variety of adult life psychiatric comorbidity. This association between FS and monoamines is required to be taken into consideration in the therapeutic protocols that target epileptic patients. Dreier and his coworkers (46) reported that in children with three or more FS admissions, the risk of psychiatric conditions was higher than in children with none. It is found that epilepsy risk was especially high at young ages but continued even after many decades when the risk of epilepsy at different ages is considered (47).

Depression and epilepsy have bidirectional comorbidity, meaning that persons with epilepsy are more likely to acquire depression and vice versa. Individually, each of these illnesses can be amplified by behavioral consequences that affect the quality of life, but less is known about these interactions when depression and epilepsy coexist (48). Newborns appear to be particularly susceptible to various types of brain injury (49). This might be linked to the higher levels of neurotransmitters in younger children compared to adults. As a result, external or internal abnormalities in a young brain’s normal function are likely to hinder biogenic amine production and proper brain development. Neurotransmitters and neuromodulators play a major role in the wiring of neural circuits. These chemical substances affect the formation of synapses and the construction of connections by modulating electrical activity, excitability, and neurotrophin synthesis. Moreover, high levels of those biogenic amines and/or overexpression of their receptors only occur briefly and only at certain periods (50).

## Conclusion

The present study concludes that complex FS at an immature stage of development can cause persistent variations in the cortical and hippocampal monoamine neurotransmitter (5-HT). The present results shed more light on the link between FS and the psychiatric co-morbidity that may be manifested by adults who suffered from FS at young ages. These findings could be of use in the protocols suggested for FS therapeutic interventions and in the medications that could interfere with the process of epileptogenesis. 

## Authors’ Contributions

O A, H M, N R, and R E contributed to the study conception and design. O A and H M conducted material preparation, animal breeding, data collection, and analysis. The first draft of the manuscript was written by O A and all authors commented on previous versions of the manuscript. All authors read and approved the final manuscript.

The criteria for authorship are according to the ICMJE guidelines for authorship criteria and based on the Committee on Publication Ethics (COPE) recommendations.

## Conflicts of Interest

All authors declare that they have no conflicts of interest.
